# Metastatic Castration-Resistant Prostate Cancer Remains Dependent on Oncogenic Drivers Found in Primary Tumors

**DOI:** 10.1200/PO.21.00059

**Published:** 2021-09-16

**Authors:** David J. Einstein, Seiji Arai, Carla Calagua, Fang Xie, Olga Voznesensky, Brian J. Capaldo, Christina Luffman, Jonathan L. Hecht, Steven P. Balk, Adam G. Sowalsky, Joshua W. Russo

**Affiliations:** ^1^ Division of Medical Oncology and Cancer Center, Beth Israel Deaconess Medical Center, Harvard Medical School, Boston, MA; ^2^ Department of Urology, Gunma University Hospital, Maebashi, Japan; ^3^ Laboratory of Genitourinary Cancer Pathogenesis, National Cancer Institute, Bethesda, MD; ^4^ Department of Pathology, University of Massachusetts Medical Center, Worcester, MA; ^5^ Department of Pathology, Beth Israel Deaconess Medical Center, Boston, MA

## Abstract

Metastatic prostate cancer is initially sensitive to androgen receptor inhibition, but eventually becomes castration-resistant prostate cancer (mCRPC). Early use of more intensive therapies targeting androgen receptor and other oncogenic drivers in treatment-naïve primary prostate cancer (PC) may be more effective than that in advanced mCRPC. However, analysis of primary tumors may not reveal targetable metastatic drivers that are subclonal in the primary tumor or acquired at metastatic sites.

**METHODS:**

PC samples spanning one patient's clinical course: diagnostic biopsies, pre- or post-enzalutamide metastatic biopsies, and rapid autopsy samples including a patient-derived xenograft (PDX) were analyzed by targeted exome sequencing followed by phylogenetic analysis.

**RESULTS:**

Left- and right-lobe primary PC tumors appeared to diverge, with the right acquiring additional shared mutations and striking differences in copy number alterations that later appeared in metastatic samples during the treatment course and at autopsy, whereas the left base tumor maintained a quiet copy number alteration landscape and partitioned into a dead-end node. *RB1* loss, a common finding in advanced castration-resistant disease, was identified throughout mCRPC samples, but not in the primary tumor. Significantly, a truncal EGFR-activating mutation (R108K) was identified in the primary tumor and was also found to be maintained in the mCRPC samples and in a PDX model. Furthermore, the PDX model remained sensitive to the EGFR inhibitor erlotinib, despite the presence of both *RB1* and *BRCA2* losses.

**CONCLUSION:**

These findings indicate that truncal alterations identified in primary PC can drive advanced mCRPC, even in the presence of additional strong oncogenic drivers (ie, *RB1* and *BRCA2* loss), and suggest that earlier detection and targeting of these truncal alterations may be effective at halting disease progression.

## INTRODUCTION

Localized prostate cancer (PC) can be curable, but metastatic recurrence is unfortunately common. Metastatic PC is initially sensitive to androgen receptor (AR) inhibition, but eventually becomes castration-resistant (mCRPC). Early use of more intensive therapies targeting AR and other oncogenic drivers may be more effective than in advanced mCRPC. However, as oncogenic alterations found in mCRPC are less frequent in treatment-naïve primary PC, analysis of primary tumors may not reveal targetable metastatic drivers that are subclonal in the primary tumor or acquired at metastatic sites. Here, we analyzed PC samples spanning one patient's clinical course: diagnostic biopsies, pre- or postenzalutamide metastatic biopsies, and rapid autopsy samples including a patient-derived xenograft (PDX). Consistent with mCRPC being driven by nontruncal alterations, we identified *RB1* loss throughout mCRPC samples, but not in the primary tumor. However, we identified a truncal epidermal growth factor receptor (EGFR)-activating mutation (R108K) in the primary that was maintained in mCRPC samples and the PDX and significantly showed that the PDX still responded to the EGFR inhibitor erlotinib despite the presence of both *RB1* and *BRCA2* losses. These findings indicate that truncal alterations identified in primary PC can drive advanced mCRPC, even in the presence of additional strong oncogenic drivers (ie, *RB1* loss and *BRCA2* loss), and suggest that earlier detection and targeting of these truncal alterations may be effective at halting disease progression.

**CONTEXT**

**Key Objective**
Can targeted sequencing of hormone-sensitive primary prostate cancer (PC) identify drivers of metastasis that are targetable in advanced castration-resistant prostate cancer (CRPC)?
**Knowledge Generated**
Our findings indicate that truncal alterations identified in primary PC (ie, EGFR R108K) can drive advanced metastatic CRPC, even in the presence of additional strong oncogenic drivers (ie, *RB1* loss and *BRCA2* loss). Furthermore, the EGFR (R108K) truncal alteration can be effectively targeted with available therapies to inhibit tumor growth, despite the presence of additional drivers.
**Relevance**
These results suggest that earlier detection and targeting of truncal alterations may be effective at halting disease progression and support the biopsying and clinical sequencing of hormone-sensitive disease from primary PC to identify targetable drivers of advanced CRPC.

## RESULTS

Previous autopsy studies have demonstrated the monoclonal origin of metastatic PC, with oncogenic driver alterations being relatively conserved across metastatic sites.^1-3^ However, most studies have inferred cancer evolution from autopsy specimens alone, without access to longitudinal tissue specimens. Here, we investigate whether oncogenic drivers of advanced disease are present in the primary tumor, thus allowing for early identification of therapeutic targets without metastatic tissue, especially in men experiencing biochemical recurrence (rising serum prostate–specific antigen, without overt metastases) or men who have undergone prostate biopsy and then are discovered to have metastatic disease on staging imaging.

**FIG 1.**
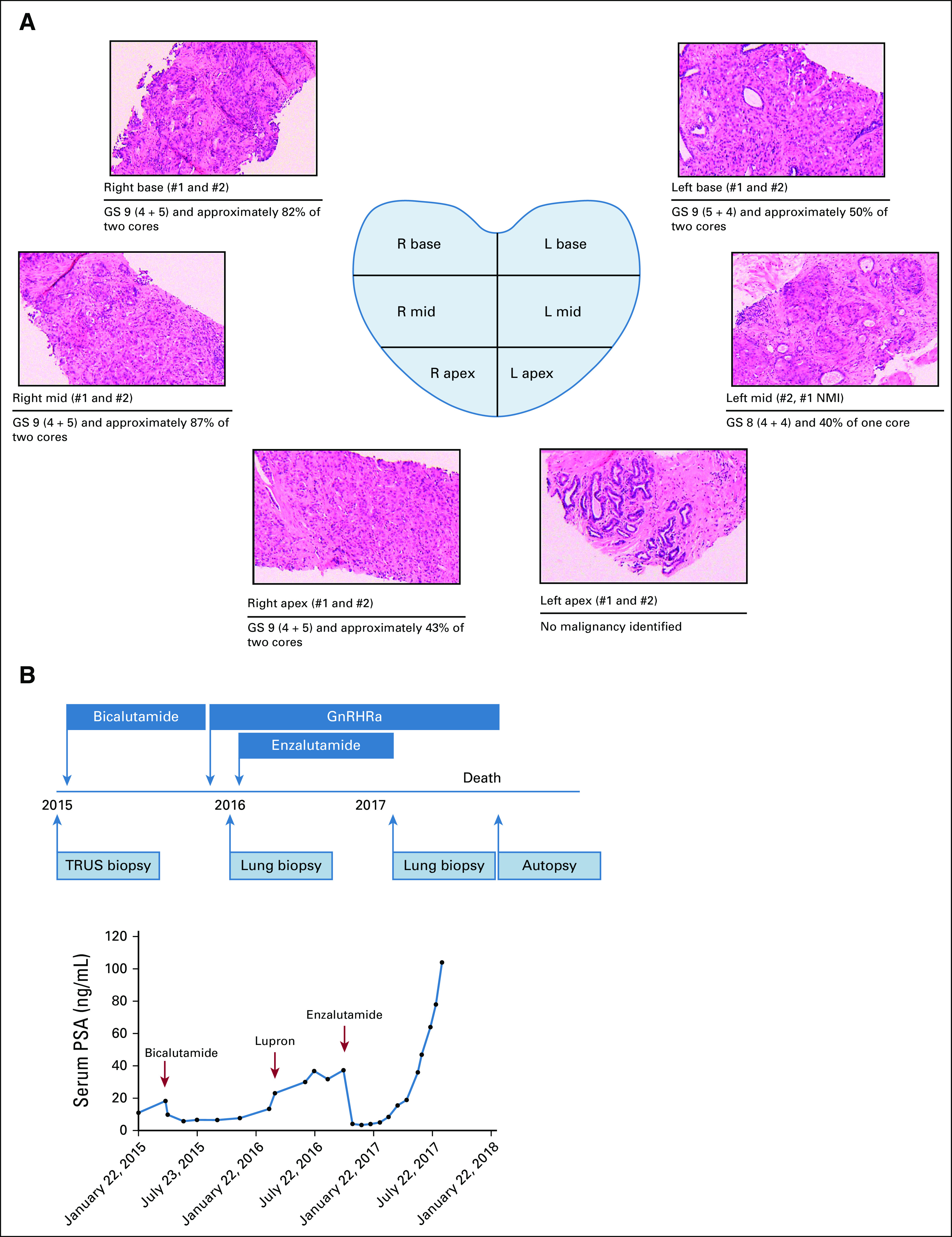
Clinical course and pathologic assessment of primary prostate cancer in rapid autopsy patient. (A) Representative H + E stains of biopsy cores sampled from the six regions of the prostate. Two cores were taken per region, and the cumulative GS and percent of core involvement are described. Prostate diagram created with BioRender.^4^ (B) (Upper) Timing of patient treatment, clinical biopsies, and rapid autopsy and (Lower) PSA responses following multiple androgen deprivation therapies. GS, Gleason score; GnRHRa, gonadotropin-releasing hormone receptor agonist; PSA, prostate-specific antigen; TRUS, transrectal ultrasound biopsy.

Following an elevated screening of prostate-specific antigen (PSA), a 68-year-old Black gentleman underwent prostate biopsy, yielding 9 of 12 cores with Gleason score (GS) 4 + 4, 4 + 5, and 5 + 4 adenocarcinoma (Fig 1A). Imaging revealed pathologic pelvic and para-aortic lymphadenopathy and bone metastases. He received bicalutamide monotherapy given his preference to avoid androgen deprivation therapy (ADT) toxicity, followed 11 months later at progression, by conventional ADT with a gonadotropin-releasing hormone receptor (GnRHR) agonist (per contemporary standard of care; Fig 1B). After only five months of ADT response, he consented to a clinical trial of enzalutamide with tumor biopsies acquired before therapy initiation and at progression, which were both obtained from a lung metastasis. He progressed after six months with significant dysgeusia and dysphagia, declined further therapy, and died seven months later. We performed a rapid autopsy, for which he previously consented, and collected 15 additional tumor samples from lung, liver, lymph nodes, and bone metastases.

We isolated DNA from diagnostic prostate biopsies (including pathologist-assessed benign areas for matched normal DNA) and matched lung metastasis biopsies pre- and post-enzalutamide therapy, rapid autopsy samples, and two PDXs created from a liver metastasis. We performed targeted sequencing using a single custom hybrid capture panel of 754 genes altered in cancer generally^5^ or PC specifically,^6^ including baits against important intronic regions in key genes: *AR*, including its upstream enhancer,^7,8^ *ERG* (introns 3-4), *ERBB2*, *RB1*, *TMPRSS2*, and *TP53* (Data Supplement). We analyzed somatic mutations and copy number alterations (CNAs), and we modeled cancer evolution using a phylogeny prediction algorithm. Notably, deleterious germline alleles of tumor suppressors were not observed. Data are available in the database of Genotypes and Phenotypes (dbGaP, accession: phs002398.v1.p1), and a Genomic Supplement with Variant Call Format (VCF) segmentation (SEG) files are available on GitHub.^9^

**FIG 2.**
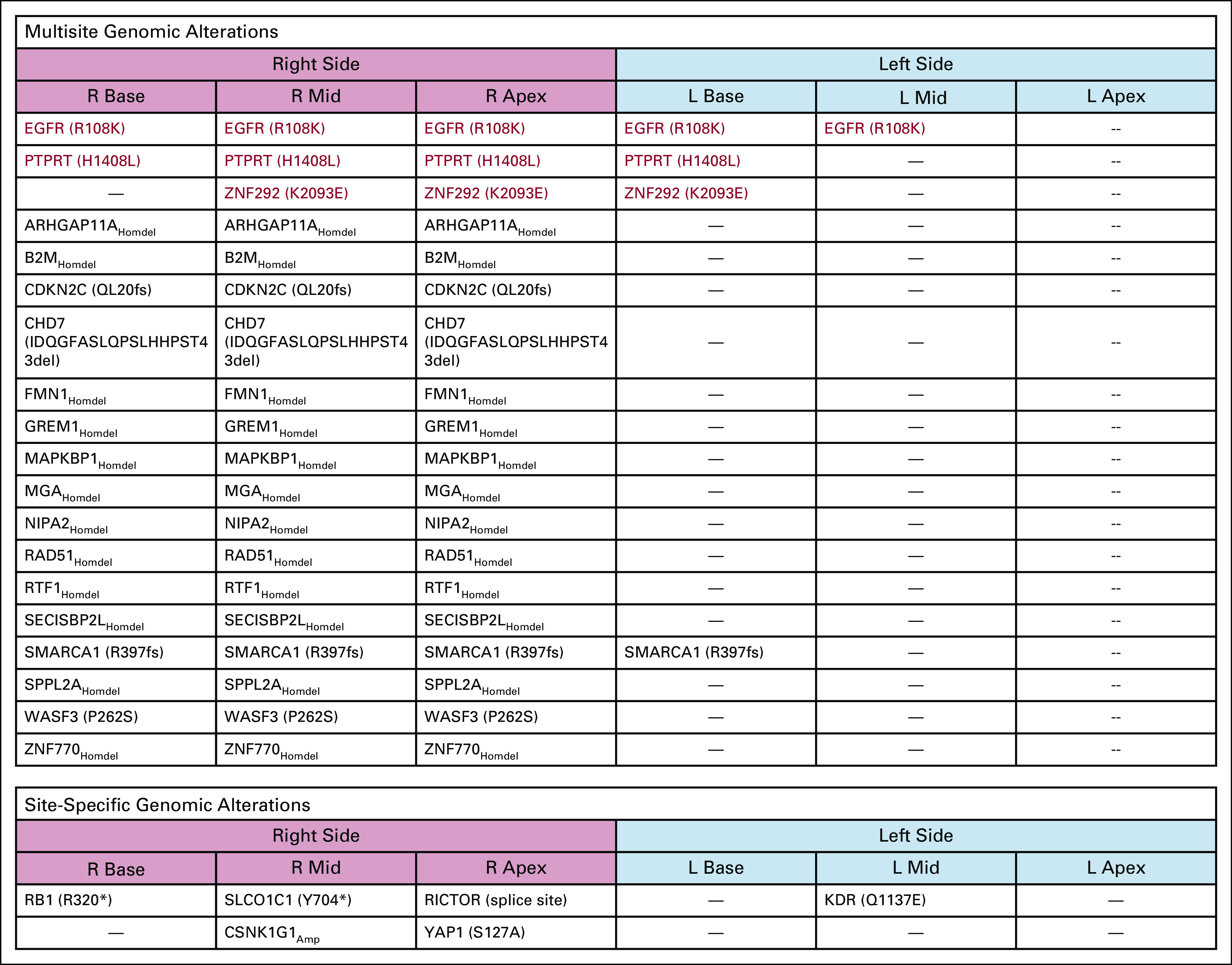
Genomics of primary prostate cancer diagnostic biopsies. (Upper) Multisite genomic alterations and those regions of the prostate in which they were found. Alterations listed in RED were found in biopsied tumor on both the right and left side of the prostate. (Lower) Site-specific genomic alterations are those alterations found only in biopsy cores from specific regions. Copy number alteration calls are based on estimates from Genome Identification of Significant Targets in Cancer (GISTIC) version 2.

**FIG 3.**
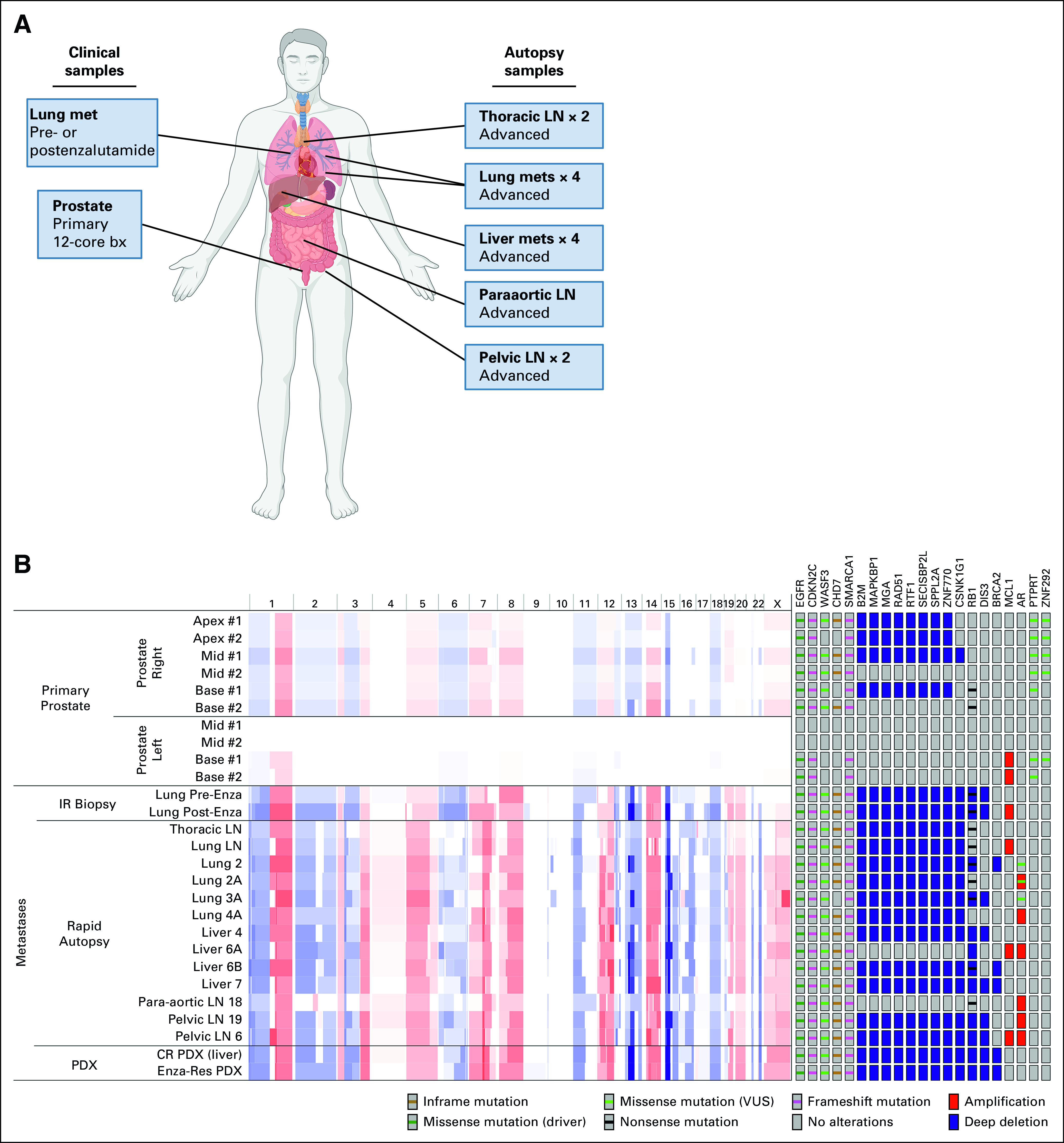
Genomics of primary advanced metastatic PC biopsies. (A) Breakdown of those tumor biopsies obtained during clinical care and those obtained during research rapid autopsy. (B) (Left) IGV plot showing chromosomal copy number variation across all sequenced tumor samples. The top 10 tracks are primary PC samples split into right- and left-sided samples, followed by 15 tracks showing advanced metastatic samples derived from clinical biopsies (Lung Pre-Enza Bx and Lung Post-Enza Bx) or samples obtained at rapid autopsy (remaining metastatic samples). The last two tracks are BID-PC5-CR PDX tumors derived from a liver met. Red indicates copy number gains, and whole blue indicates copy number losses. (Right) Genomic alterations significantly enriched in either the primary tumor samples or the advanced metastatic samples. Copy number alteration calls are based on estimates from GISTIC. For *P* values associated with specific genomic alterations, refer to the Data Supplement. Human figure with organs created with BioRender.^4^ bx, biopsy; GISTIC, Genome Identification of Significant Targets in Cancer (GISTIC) version 2; IR, interventional radiology; LN, lymph node; met, metastasis; PC, prostate cancer; PDX, patient-derived xenograft; VUS, variant of uncertain significance.

Broadly, there were evidence of a monoclonal origin of tumor in the prostate given shared mutations [EGFR (R108K), PTPRT (H1408L), and ZNF292 (K2093E)] across all diagnostic biopsies (Fig 2 and Data Supplement) and evidence of whole-genome doubling (WGD) by allele-specific copy number analyses and two copies of the X chromosome in almost every tumor sample. WGD is an infrequent event in primary PC.^10^ However, the left- and right-lobe tumors appeared to diverge, with the right acquiring additional shared mutations and striking differences in CNAs that later appeared in metastatic samples during the treatment course and at autopsy, whereas the left base tumor only developed a more focal chromosome 1q gain (Fig 2 and Figs 3A and 3B). Notably, despite containing high-grade (GS 5 + 4 and 4 + 4) PC, with a particularly aggressive intraductal pattern, the left base tumor had a relatively quiet CNA landscape (Fig 3B and Genomic Supplement),^9^ and phylogenetic analysis of CNAs and mutations with consideration of allele-specific somatic copy number alterations and variant allele frequency indicated that the left-lobe tumor partitioned into a dead-end node and did not seed the majority of metastases (Data Supplement). This supports an association between quiet CNA landscape and indolence and indicates that additional nontruncal genomic events found in the right-sided tumor were required to facilitate metastasis and treatment resistance.

Furthermore, phylogenetic analysis partitioned those autopsy samples from the lymph nodes into truncal branches (Data Supplement, right prostate and node 1), whereas visceral metastases to the lung and liver partitioned to separate branches with acquisition of additional genomic alterations (Data Supplement, Nodes 2 and 3). Clinically, visceral metastatic PC is known to act more aggressively than metastases to lymph nodes or bone without visceral involvement, a distinction made in several clinical trials.^11,12^ This analysis suggests an association between genomics and metastatic seeding: on top of the homozygous deletions of tumor suppressors and amplification of *AR* seen in nodal metastases, lung involvement was defined by mutations in *AR* and *ITSN1* and liver involvement was defined by mutations in genes involved in epigenetic regulation, *ARID5B* and *HIST1H1C*. Alterations could have particular importance in *ARID5B*, which has been described as a positive regulator of AR transcription.^13^

The EGFR (R108K) mutation was present in all metastatic sites and showed increased variant allelic frequency when comparing left prostate with right prostate and comparing right prostate with metastases, supporting its role as a truncal driver mutation (Fig 3B and Data Supplement). Although not previously reported in PC, this mutation has been found in glioblastoma; it occurs in the extracellular domain and results in increased basal EGFR phosphorylation targetable by erlotinib.^14,15^ This is distinct from the activating kinase domain mutations seen in lung adenocarcinoma. PTPRT is a phosphatase that interacts with EGFR,^16^ and the novel H1408L mutation resides in a region wherein mutations may decrease phosphatase activity and thus potentially further activate EGFR.^17^ Other novel mutations acquired in the right prostate were present in all (*CDKN2C*, *WASF3*, and *SMARCA1*) or nearly all (*CHD7*) metastatic sites and PDXs.

Metastases were also enriched for deletions in a number of genes all derived from the right prostate within 15q11-21 (Fig 3B and Data Supplement). In particular, *B2M* loss has been described as a mechanism of resistance to programmed death-1 (PD-1) inhibition in melanoma and HLA class I antigen–processing machinery alterations more broadly have been associated with early disease recurrence in PC,^18-20^ raising the possibility that this defect resulted in loss of immune surveillance, even without the selective pressure of immune checkpoint inhibition. Also notable was the *RAD51* deletion, a predictive biomarker for response to poly (ADP-ribose) polymerase inhibitors.^21-23^

Consistent with previous reports, biallelic alterations to *RB1* (homozygous deletion or truncation, R320* with single-copy loss) were enriched in advanced metastatic samples (primary: 2 of 10 samples, metastatic: 14 of 15 samples, Fisher's exact test *P* = .001, Fig 3B, Data Supplement). Notably, *RB1* deep deletions were only found in metastatic sites (10 of 15 samples), and in 3 cases, they were associated with deletions of *BRCA2*, which is located in proximity to *RB1* on chromosome 13. However, *RB1* was still functionally lost in the right prostate subclone because of a truncation mutation in one allele and single-copy loss in the other. Evidence of WGD appeared before metastasis, as described above, generating two copies of the mutated allele, present in some metastases, and both copies are lost in others. Thus, although two-copy loss of *RB1* is rare in primary PC, other pathways may lead to its functional loss, which, in turn, is associated with metastatic potential.

Amplification of *AR* or its upstream enhancer occurs in approximately 81% of castration-resistant prostate cancer (CRPC).^24^ Although only six metastases had GISTIC-called AR amplifications, all metastases exhibited broad gains across the AR locus including the upstream enhancer (Data Supplement). Significantly, four metastases (Liver 4, Liver 6A, Pelvic LN 19, and Pelvic LN 6) had amplifications extending from approximately 800 kb upstream of the locus to within introns 3, 4, and 6 and encoding the AR hinge region and ligand-binding domain. This could represent a genomic rearrangement in the amplified regions facilitating expression of an AR variant lacking the ligand-binding domain. Concurrent AR rearrangements and amplifications occur in approximately 20% of CRPC, leading to increased AR-V4, AR-V7, and AR-V9 expression.^25^

We used matched biopsies of a lung metastasis pre-enzalutamide and at progression to analyze resistance mechanisms (Data Supplement). Surprisingly, both pre- and post-enzalutamide samples exhibited two-copy deletion of *RB1*, suggesting that the broad *RB1* loss in most metastases occurred before enzalutamide exposure, not in response. The post-enzalutamide sample showed alterations in several receptor tyrosine kinases (RTKs), including further gains to *NTRK1* and *DDR2* and a novel R1118Q mutation in *KDR,* also known as *VEGFR2*, occurring in the kinase domain where gain-of-function mutations are described (Data Supplement).^26,27^ Further gains to *MCL1* also appeared. We hypothesize that this bicalutamide-pretreated tumor had relative enzalutamide resistance associated with baseline *RB1* loss and quickly developed increased RTK signaling and suppressed apoptotic response (*MCL1* gains) on enzalutamide. *NTRK1*, *DDR2*, and *MCL1* are all located on chromosome 1q and are frequently coaltered in CRPC (*MCL1* coamplification in 22 of 22 *NTRK1*-amplified tumors, *P* < .001, q < 0.001 and in 22 of 27 *DDR2*-amplified tumors, *P* < .001, q < 0.001).^28-30^ Evidence of a focal amplification comprising chromosome 1q21-23 on top of pre-existing arm-level gains of 1q originating in the primary tumor is present in nearly all metastasis samples (Genomic Supplement).^9^

Liver metastasis tissue was transplanted subcutaneously into immunocompromised male mice (intact or castrated), and a PDX emerged in one of the castrated mice (BID-PC5-CR). This castration-resistant PDX had a transient partial response to enzalutamide (Data Supplement). Both the castration-resistant and subsequent enzalutamide-resistant PDXs had similar genomic alterations as the liver metastases and partitioned with the liver metastases in the phylogenetic analysis (Fig 3B and Data Supplement). Relative to BID-PC5-CR, the derived enzalutamide-resistant PDX demonstrated one new mutation *AXIN2* (*Y187D*), increased loss of *TSC1*, *RAB14*, *ABL1*, and *SETX*, and further enrichment of gains to *MET*, *CDK6*, *PIK3CG*, and *FOXA1*, among others that might have contributed to resistance (Data Supplement). Importantly, these gained genomic alterations were distinct from those found in the post-enzalutamide lung biopsy.

**FIG 4.**
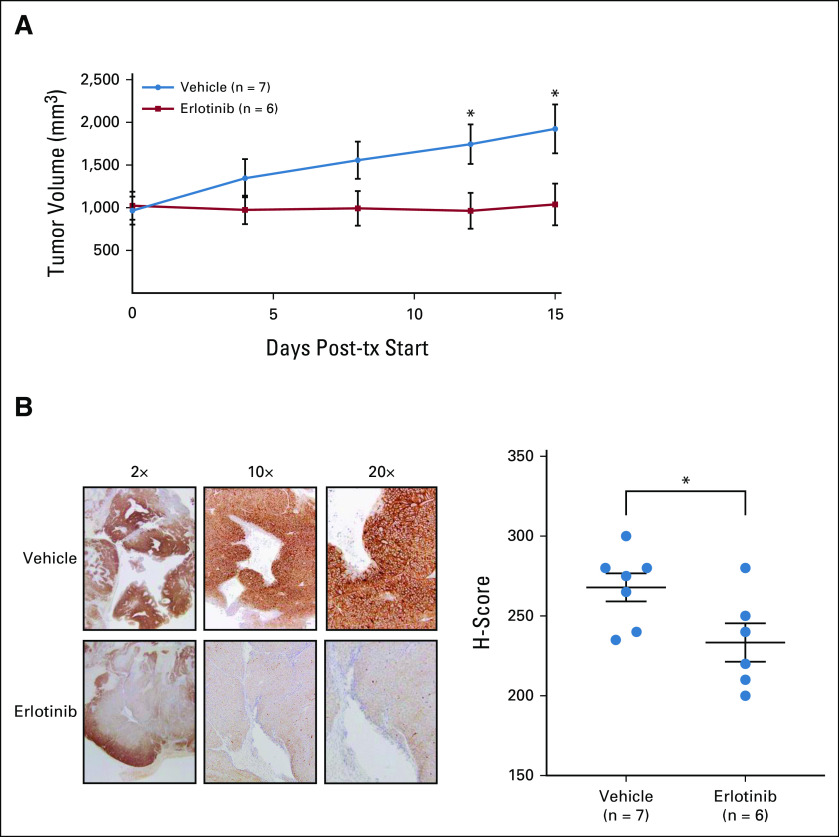
EGFR (R108K) drives patient-derived xenograft growth and is sensitive to erlotinib. (A) BID-PC5-CR patient–derived xenografts were established in castrated, immunocompromised mice and then treated with daily oral gavage of vehicle or erlotinib (100 mg/kg/day once a day) for 15 days, *P < .05, Mann-Whitney U test, two-tailed. Error bars represent SEM. (B) (Left) Immunohistochemistry for pEGFR (Y1173) performed on FFPE sections of tumor and (Right) quantification of pEGFR (Y1173) staining by H-score, *P < .05, unpaired *t* test, two-tailed. Error bars represent SEM. FFPE, formalin-fixed paraffin-embedded; tx, treatment.

Although the BID-PC5-CR PDX harbored the truncal *EGFR* mutation, it did not harbor the *PTPRT* mutation that we speculated may further enhance EGFR activity (Fig 3B). Moreover, relative to the primary tumor, it maintained additional potent oncogenic alterations found in the metastases, including *RB1* loss. Therefore, we next tested whether the EGFR mutation was a targetable driver in this PDX. Erlotinib significantly inhibited the growth of BID-PC5-CR tumors by 48% versus control (Fig 4A, Mann-Whitney U test *P* < .05), which was associated with decreased phosphorylation of EGFR (Y1173) in erlotinib-treated tumors versus control (Fig 4B).

In summary, longitudinal tissue assessment and rapid autopsy samples revealed genomic alterations associated with metastasis—or indolence—and treatment resistance and highlighted the presence of both truncal driver alterations and acquired alterations emerging over the treatment course. One recent report also correlated autopsy tissue with longitudinal samples,^31^ and our results support the conclusions that divergent lineages spatially coexist, that one dominant lineage of several intraprostatic lineages seeds most—if not all—subsequent metastases, and that subsequent heterogeneity arises within the metastatic lineage, an evolutionary bottle neck. In addition, this case demonstrates the importance of a truncal *EFGR* (*R108K*) mutation occurring throughout the primary and metastatic sites. Furthermore, we were also able to characterize a very high-grade (GS 5 + 4) primary subclone that surprisingly did not appear to seed metastases, on the basis of CNA comparison and phylogenetic analysis. The indolent behavior of this left base tumor was associated with a quiet CNA landscape. Conversely, an additional primary subclone with acquisition of deep CNA losses in key genes (*B2M*, *RAD51*, etc) arose on the right side of the prostate and seeded all sampled metastatic sites. Early on, it lost *RB1* through mutation of one allele and loss of the other, followed by WGD, whereas treatment-resistant metastases acquired two-copy *RB1* loss. In a subset of metastases and in the BID-PC5-CR PDX, *RB1* loss was accompanied by *BRCA2* loss, a phenotype associated with particularly aggressive behavior.^32^ Visceral involvement was defined by additional alterations compared with nodal metastases. Despite dual *RB1*/*BRCA2* loss, we found that the BID-PC5-CR was responsive to EGFR inhibition with erlotinib, indicating that subsequent advanced mCRPC was still EGFR-dependent despite its acquisition of multiple additional potent oncogenic events. These findings show that early genomic testing of primary PC can identify truncal driver alterations that remain targetable vulnerabilities in metastatic sites, despite acquisition of additional potent oncogenic events. This suggests that early analysis of primary tumors and subsequent biomarker-selected targeted therapies to treat men with biochemical recurrence, or men who present with de novo metastatic disease, may prevent or markedly delay disease progression. In addition, genomic markers may define intrapatient subclones of varying aggressiveness, differentiating those with metastatic potential from those without and those with visceral involvement from nodal involvement.
